# Evaluation of Bacterial Contamination in Dressed Chickens in Lusaka Abattoirs

**DOI:** 10.3389/fpubh.2019.00019

**Published:** 2019-02-19

**Authors:** Prudence Mpundu, Allan Rabson Mbewe, John Bwalya Muma, Jessy Zgambo, Musso Munyeme

**Affiliations:** ^1^Ministry of Health, Chainama Hills College Hospital, Lusaka, Zambia; ^2^Department of Environmental Health, School of Public Health, University of Zambia, Lusaka, Zambia; ^3^Department of Disease control, School of Veterinary Medicine, University of Zambia, Lusaka, Zambia; ^4^Department of Epidemiology Biostatistics, School of Public Health, University of Zambia, Lusaka, Zambia

**Keywords:** bacterial contamination, abattoirs, dressed chickens, *Escherichia coli*, *Salmonella*

## Abstract

**Objective:** The aim of this study was to evaluate bacterial contamination and the risk factors associated with contamination of poultry during processing. Despite the rapid growth of the poultry industry, the presence of high levels of pathogenic bacteria contaminants, such as *Escherichia coli* and *Salmonella*, pose serious public health concerns in dressed chickens. These infections negatively affect the product's shelf life.

**Methods:** A cross sectional design was used to study two main poultry abattoirs in Lusaka. The processing line was used to collect biological samples along with the acquisition of risk-associated data using a structured questionnaire. Data collected both from biological sources and the risk analysis were entered into Excel and analysed in STATA version 14 for windows.

**Results:** *Escherichia coli* and *Salmonella* contamination was detected in 70 and 2.5% of the selected dressed chickens (*n* = 80), respectively. The number of total coliforms and *Escherichia coli* were observed to be significantly higher in samples from washed carcasses than pre-washed carcasses (65 and 35%, respectively). In addition, this study revealed that among the anthropogenic and exposure risk factors, bacterial contamination levels resulted mainly from a lack of hygienic practices. This included hand washing and an increased frequency of slaughters per day (>15,000).

**Conclusion:** This study indicates that the water used for dressing chickens is probably the major cause of high levels of cross-contamination. The results also highlight the issues that need to be addressed to improve environmental and carcass hygiene in a poultry abattoir.

**Significance:** Critical findings in this study are that contamination sources may be variable and hygienic practices may play a major role. In this particular study, the reuse of contaminated water was a case in point. Accordingly, there is need for both the water source and the water being used for processing to be tested.

## Introduction

Chicken is one of the highly consumed meat products across the globe, both in developed and developing nations. Global Livestock Counts report that there are almost 19 billion chickens in the world (1), making it the most common species of birds. Europe consumes an average of 2.5 kg of chicken per capita per year whereas Africa's annual average consumption per capita stands at 6 kilograms (2).

The annual broiler meat consumption in Zambia per capita is 4.8 kg, with an estimated national consumption of 62.9 million kilograms. This makes the annual production rate 81.4 million kilograms (3). In most countries, poultry is considered to be amongst the most affordable species that is slaughtered at home by most farmers and households (2). In addition, poultry meat has few religious restrictions compared to other domesticated animal species (4).

However, because of an absence of stringent hygienic practices most poultry is contaminated along the process line from primary, through secondary, to the final product (5). Bacterial microorganisms of particular importance to public health, such as coli-forms, especially *Salmonella* and *Escherichia coli* (*E. coli)*, have been found as part of the normal flora in the gastrointestinal tracts of several domestic animals, including chickens. These bacterial coli-forms are leading causes of food-borne diseases worldwide (6, 7). *Salmonella* and *E. coli* are major causes of both acute and chronic food-borne diseases in poultry and humans worldwide (7).

Compared with other animal species, cases of *Salmonella* have been well-documented in poultry products (8). The prevalence of *Salmonella* in dressed chickens has been linked to poor farming practices. *Salmonella* infections can also be acquired from other sources, such as untreated raw milk (9) or untreated water (10), but consumption of poultry meat, mainly fresh chicken meat, remains the major risk factor for acquiring infections. Different prevalence rates have been documented in several countries; in Portugal *Salmonella* prevalence rates as high as 70% have been recorded (11).

Several studies have tried to document the prevalence of *Salmonella* in dressed chickens. These studies have found that the proportion of chickens infected with *Salmonella* was 4.2% in the greater Washington DC, USA area (12); whereas a study in Nepal found the rate to be 14.5% (13, 14). All these findings were for *Salmonella* in dressed chickens. In South Africa, a further comparative study in both freshly slaughtered and frozen chickens found a prevalence rate of 19% in freshly slaughtered chickens and 11% in frozen chicken products (15). An earlier study in Zambian abattoirs found a prevalence of 20.5% in dressed chickens (16), whilst in Sudan a higher prevalence of 44.4% was recorded (17).

*E. coli*, like *Salmonella*, is a bacterium that lives in the intestines of both humans and animals. It is usually used as an indicator of facal contamination arising mostly from human facal contamination of either water sources or food. The presence of *E. coli* in chickens is an indication of poor hygienic practices in abattoirs or trading areas. A wide variety of both plant and animal foods are potential sources of *E. coli* contamination, especially chickens. *E. coli* has also been found worldwide in poultry meat products (18). The prevalence of this bacterium in both poultry and poultry products differs across different parts of the world. The prevalence rate of *E. coli* has been recorded to be as high as 98% in India (19). In Sudan, the prevalence of *E. coli* was as high as 57.8% (19), whilst in Morocco it has been reported to be 48.4% (20) and 16% in Nigeria (18).

The above findings for both *E. coli* and *Salmonella* contamination in chickens from different countries around the world reflect the various different levels of hygienic practices and quality control systems. Countries with good hygienic and manufacturing practices tend to record lower contamination levels in their slaughtering processes. The seemingly high proportion of contamination in chickens creates the impression that chicken meat is a food of significant public health concern. This is because it is a source and medium for possible food borne infections and illnesses in man (21). Annually, millions of people globally suffer from food-borne illnesses because of different food consumption patterns, including the consumption of chicken meat (22). Therefore, reducing the contamination levels in raw chickens at the different processing stages can have a significant impact on reducing the incidences of illnesses that are linked to the consumption of contaminated poultry products (23).

In most countries, including Zambia, diarrheal diseases are among the top ten leading causes of out-patient visits at clinics and hospitals (22). *Salmonella* species and *E. coli* are potentially pathogenic organisms in humans and animals. Typhoid and other diseases caused by *Salmonella* enteritidis are a common occurrence in most African Countries (22). This was exemplified in reports, such as one from 2009, where the consumption of contaminated poultry resulted in 155,000 deaths and 80.3% of these could be directly linked to the consumption of contaminated poultry (22). However, chicken is considered to be a cheaper source of animal protein by the majority of Zambians and it is among the popular food of choice in most homes, restaurants, and fast food outlets (2). Given the above scenario, this study was formulated with the main aim of assessing the levels of bacterial contamination in dressed chickens processed in abattoirs within Lusaka the capital city of Zambia. Furthermore, this study aimed to gather information linked to risk factors that may be linked to bacterial contamination in abattoir settings.

## Materials and Methods

The study design was cross sectional in nature and was conducted in Lusaka Zambia. Lusaka is located at 15°25′ south of the equator and 28°17′ east of Greenwich. The study was carried out from November 2016 to March 2017. Out of four possible choices, we selected two abattoirs for this study because of ease of accessibility and the volume of chickens they each produced. Furthermore, the remaining two abattoirs were not included because one of the abattoirs was undergoing renovation and the other abattoir did not grant us permission to conduct the study. The two selected abattoirs are identified here as Abattoirs A and B. The sampling cohort consisted of only dressed chickens that were slaughtered at the abattoir during the period of the study. A circular systematic random sampling method was used to pick a chicken for swabbing. The sample size calculation for questionnaires was estimated using 57.8% prevalence from a previous study of *E. coli* contamination of chicken carcasses in abattoirs (24). A sample size of 174 was required to detect a 30% reduction at 80% power for a two-sided 5% level of significance using a Pearson chi-square test. A minimum of 87 food handlers were interviewed at each abattoir along with one person from among the abattoir management staff. The sample size for bacteriological analysis was calculated according to the food inspection manual of the Food and Drugs Act Cap 303 2009 using the daily abattoir throughputs. The abattoir throughputs were 8,000 and 20,000 birds per day for Abattoirs A and B, respectively. According to the inspection manual, the recommended sample size using this range was five dressed chickens per batch. A batch at the two abattoirs was defined as chickens that came from the same flock and had homogenous characteristics (i.e., the same owner, the same farm, etc.) with the total number being 1,000 birds per day. The two abattoirs made up the target population (*N*), from which a sample population (*n*) was drawn. As part of the quality control for water being used in an abattoir, water samples were collected before the carcasses were washed and immediately after carcass washing. These samples were also taken to the laboratory for analysis alongside the biological samples.

A total of 80 carcasses were randomly selected using the circular systematic random sampling system for each batch by selecting five dressed chickens. At the end of the sampling period in the two abattoirs, 80 carcasses were selected for bacteriological examination from three different batches representing three poultry farms.

### Sample Collection and Processing

A two-stage sampling process was used in this study. Sampling was performed immediately after evisceration and after washing before packaging using a sterile metal string to outline a 10 cm^2^ area of the dressed chicken carcass. The outlined area was swabbed with sterile cotton gauze wrapped around the end of a flat swab-stick. The swabs were then transferred into a tube containing transport media (Cary-Blair Transport Media). Each tube containing the carcass swab was marked by sequential numbering according to the batch, date, and whether it was prior to wash or after wash. The water samples were collected using a sterile scoop and poured in a sterile sampling bottle which was labelled accordingly. All samples collected were then transported promptly in an ice-cold cooler box, equipped with ice packs, to the Microbiology Disease Control Laboratory in the School of Veterinary Medicine at the University of Zambia. All samples were cultured on the day of collection, and before culture in the laboratory they were maintained at 0–4°C. In the laboratory, the swabs were removed from the transport media and placed in test tubes containing 0.5% peptone water and mixed using a vortex mixer.

### Isolation and Identification of *Salmonella*

This was performed by inoculating each sample into 9.0 mL of sterile peptone water in screw-capped test tubes. Following this, the test tubes were incubated at 37°C for 3 hrs to allow for resuscitation of the bacteria. Thereafter, a loop full of the peptone broth culture was transferred to *Salmonella* enrichment Rappapport broth and incubated at 44°C for 48 hrs. Following this, a loop full of the broth culture was streaked onto Xylose Lysine Deoxycholate (XLD) agar followed by incubation at 37°C for 24 h. Presumptive *Salmonella* colonies with black centres on XLD agar were picked and sub-cultured on Nutrient agar at 37°C for 24 h. Presumptive *Salmonella* colonies were confirmed biochemically by inoculating into Triple Iron Sugar, urea, citrate and Sulphide Indole Motility (SIM) medium (17).

A total bacterial count was performed by placing each sample swab in sterile 0.5% peptone water, and then incubated for 3 h at 37°C. A serial dilution, from 10^1^- to 10^3^-fold, was prepared from each sample broth culture. Each dilution (1.0 mL) was transferred to sterile Petri dishes in duplicate and sterile molten agar was added using the pour plate method. The plate cultures were incubated at 37°C for 24 h. The colony forming unit (CFU) was determined from appropriate dilutions (17).

The enumeration of *Escherichia coli* was performed in Eosin Methylene Blue agar (EMB) using the pour plate method and an appropriate dilution factor. Plate cultures were incubated aerobically at 44°C for 24 h. Colonies with a distinct metallic shiny were counted as a CFU (17).

Presumptive *E. coli* colonies were similarly confirmed biochemically by inoculating into Triple Iron Sugar, urea, citrate and Sulphide Indole Motility (SIM) medium (17).

The collected water samples were also analysed for *Salmonella* and *E. coli* using the methods outlined above.

### Data Collection Techniques and Tools

Data collection was based on bacteriological sample collection for bacterial contamination, interviews using structured questionnaires for assessing risk factors such as hygiene practice, knowledge, sanitary conditions, and a checklist for triangulation.

### Statistical Analysis

A survey summary and descriptive statistics were used to examine if the various risk factors identified were associated with the presence of bacteria in abattoirs.

First, a univariate analysis by way of cross-tabulations using a Pearson's chi-square test was preformed and this was followed by multiple logistic regressions. A *p* < 0.05 was considered significant with a 95% confidence interval (95%CI). STATA version 14.0 (Stata Corporation, College Station, TX, USA) was used for all analyses in this study.

## Results

### Descriptors of Bacterial Prevalence

Out of 80 chicken carcasses sampled, 56 (70%) had a total coliform count contamination. *Salmonella* accounted for 2.5% of the contamination, while *E. coli* accounted for 55% (Table 1).

**Table 1:** Bacterial contamination status of chicken samples (*n* = 80).

**Bacteria**	**Number**	**%**	**95% CI**
Total coliform count (TCC)	56	70.0	58.9–79.2
*E. coli*	44	55.0	43.8–65.7
*Salmonella*	n = 2	2.5	1.75–3.80

### Comparative Assessment Between Abattoirs A and B

A chi-square analysis comparing the two types of abattoirs and *E. coli* contamination revealed that Abattoir B had the highest contamination level, with 77.5%, of chicken carcasses being contaminated with *E. coli*. In contrast in Abattoir A only 32.5% of the chicken carcasses were contaminated with the same bacteria (Table 2).

**Table 2:** Association of dressed chicken contamination with different sampling sites (*n* = 80).

**Site**	***E. coli*** **contamination**			**χ^2^**	***P-*value**
	**Present (%)**	**Absent (%)**	**Total (%)**		
Abattoir A	13 (16.3)	27 (33.8)	40 (50.0)		
Abattoir B	31 (38.8)	9 (11.3)	40 (50.0)	16.3636	<0.001
	44 (55.0)	36 (45.0)	80 (100.0)		

### Bacterial Contamination at Two Processing Points at the Abattoirs

When bacterial type was adjusted based on the swabbing time point*, E. coli* contamination was 65% in post-washed chicken carcasses and 35% in pre-washed chicken carcasses.

### Bacterial Contamination of Water Samples

Bacterial contamination was also assessed in the different water samples. *E. coli* contamination was found in water samples after carcass washing and was absent in water samples before carcass washing.

### Assessment of Abattoir Risk Factors for Bacterial Contamination

A univariate analysis showed that a high daily dressing frequency was found to be significantly related to contamination level (*p* < 0.001), as well as availability of hand washing soap in the abattoir (*p* < 0.001), source of water (*p* < 0.001), a lack of training on chicken handling (*p* < 0.001), lack of inspection of dressed chickens (*p* < 0.001), and the daily frequency of general abattoir inspections (*p* < 0.002; Table 3).

**Table 3:** Cross tabulation of variables (*n* = 174).

**Site**	**Process Throughput per day**		**Total**	*****χ^2^*****	***p-value***
	**10,000 to 15,000**	**15,000 and above**			
Abattoir A	87 (50.0)	0 (0.0)	87 (50.0)	174	0.001
Abattoir B	0 (0.0)	87 (50.0)	87 (50.0)		
			174 (100.0)		
**AVAILABILITY OF HAND WASHING FACILITIES**					
**Site**	**Availability of hand washing soap**		**Total**	***χ^2^***	***p-value***
	**Available**	**Absent**			
Abattoir A	87 (50.0)	0 (0.0)	87 (50.0)	174	0.001
Abattoir B	0 (0.0)	87 (50.0)	87 (50.0)		
			174 (100.0)		
**TRAINING**					
**Site**	**Training in food safety**		**Total**	**χ^2^**	***p*****-value**
	**Trained**	**Not trained**			
Abattoir A	87 (50.0)	0 (0.0)	87 (50.0)	29.1	0.001
Abattoir B	62 (35.6)	25 (14.4)	87 (50.0)		
			174 (100.0)		
**INSPECTION**					
**Site**	**Inspection of chickens**		**Total**	**χ^2^**	***p*****-value**
	**Inspected**	**Not inspected**			
Abattoir A	85 (48.9)	2 (1.1)	87 (50.0)	31.4	0.001
Abattoir B	56 (32.2)	31 (17.8)	87 (50.0)		
			174 (100.0)		
**FREQUENCY OF INSPECTION**					
**Site**	**Time of chicken inspection**		**Total**	**χ^2^**	***p*****-value**
	**On arrival at abattoir**	**After processing**			
Abattoir A	30 (17.2)	57 (32.8)	87 (50.0)	23.6	0.001
Abattoir B	62 (35.6)	25 (14.4)	87 (50.0)		
			174 (100.0)		

A logistic regression analysis was conducted to determine the strength of the association between the abattoir risk factors most likely to influence bacterial contamination. High process throughput per day was found to be a significant risk factor for contamination. This was evident in abattoirs processing 15,000 chickens and above per day, being four times more likely to be contaminated than those that had lesser throughputs (*OR* = 4.5; 95% CI: 1.7–11.7). The odds ratio figure remained significant even after controlling for the other risk factors (*OR* = 4.5; 95% CI: 1.7–11.7) at *p* < 0.002 under the adjusted model (Table 4).

**Table 4:** Logistic regression of bacterial contamination and risk factors at the abattoir level.

**Variables**	**Unadjusted**			**Adjusted (*****n*** **=** **130)**		
	**OR**	***p*-value**	**95% CI**	**OR**	***p*-value**	**95% CI**
**PROCESS THROUGHPUT**						
10,000 to 15,000	(ref)			(ref)		
>15,000	4.5	<0.001	1.7–11.7	4.5	<0.002	1.7–11.7
**MEANS OF HAND WASHING**						
Did not use anything	(ref)			(ref)		
Used Soap	0.0	<0.002	1.7–11.7	1		
**TRAINED IN FOOD SAFETY**						
Trained	(ref)					
Not trained	1					
**INSPECTION OF DRESSED CHICKENS**						
Inspected	(ref)					
Not inspected	3.8	<0.078	8.6–17.1			
**FREQUENCY OF INSPECTION**						
On arrival	(ref)					
On arrival and after processing	1.3	0.367	0.6–2.7			

^*^(ref) means “represents the ‘reference category' when interpreting the OR.”

## Discussion

This study combined a risk exposure assessment with biological determination of bacterial contamination in poultry abattoirs. Emphasis was heavily placed on bacterial contamination originating from processed chickens before and after washing. Both total coliform and *E. coli* were recovered in high numbers after carcass washing in the two abattoirs studied. Comparatively, pre-washed chickens, post evisceration, had lower levels of contamination at 35% compared to post-washing with higher contamination levels at 65%. These data strongly suggest that the observed increase in contamination levels after washing is likely to be due to the increased use of reused water during processing of the chicken carcasses. Consequently, good water quality and a close monitoring of water are needed throughout the processing line.

This can only be achieved by employing a stringent water management system from the source to the processing steps. This is because water is a potential vehicle for the direct transmission of bacteria in most countries, and is therefore a leading cause of disease (22).

The data show that most of the samples were beyond acceptable standards with regards to Food Standard (25). The current findings are congruent with earlier findings (17) that identified *E. coli* as the major contaminant at all critical control points compared with *Salmonella*, and this was also one of the findings in this study. Chickens are susceptible to infection by a variety of bacteria especially those that are pathogenic (26). This may partially be attributed to their rearing systems, especially the deep litter system. It allows chicken droppings to be soaked within the deep litter system allowing enteric microbial contamination within the poultry houses. However, the isolation of more *E. coli* than *Salmonella* is an index of hygienic quality especially in the hatcheries (17).

This current study recorded lower levels of *Salmonella* (2.5%) infection compared to a previous similar study in Zambia that reported a prevalence of 20.5% (16). This difference in prevalence rates can be partly attributed to differences in sample size and the characteristics of the sampled chickens used between the two studies. Lower prevalence estimates of *Salmonella* have also been reported in other countries, such as the United States of America, where it was 4.2% (14). This may partly be explained by the biological nature of the pathogen and how it is shed from the infected hosts (27).

Like *Salmonella, E. coli* was identified in this study and emerged as another major contaminant. Variations in *E. coli* contamination levels have been observed by various authors such as a prevalence of 34.5% in India (19) whereas of a prevalence of 57.8% was observed Sudan (17). There is some congruence in these findings with our current study. Similarly, our study also observed higher levels of *E. coli* and other coliforms.

Differences in *E. coli* contamination were observed between the two abattoirs. Abattoir A had a rate of 32.5% while Abattoir B had a rate of 77.5%. These differences may be linked to either environmental factors or the machinery being used in the two abattoirs: Abattoir A uses multistage scalding tanks, which have been reported by others to have significant contribution in terms of bacteria reduction as compared to the single scalding tanks being utilized by abattoir B (28). The scalding water was changed on a daily basis in abattoir A whilst in abattoir B it was changed every other day. During scalding, there is massive cross-contamination that takes place because each bird transfers bacteria to the scalding tank. Scalding tanks can only reduce bacterial contamination, such as *E. coli* and indicator bacteria like the total coliform count, if the scalding water in the tanks is continuously replaced with fresh water. The current study further revealed that the majority of food handlers in abattoir A were trained in food safety as opposed to Abattoir B, which had some food handlers that had not been trained in food safety. As earlier indicated, this causes a lapse in ensuring that the chickens are of good quality. Generally, slaughter operational hygiene, and quality management are closely related with a reduction in the overall contamination of carcasses (29). Differences in the training of food handlers between the two abattoirs may also explain the reasons for these differences in contamination levels.

Risk factors that were shown to contribute to bacterial contamination were the high frequency of dressing events per day, the availability of hand washing soap, the source of water, training in food safety, performance of inspection and the frequency of inspections. All these factors were significant (*p* < 0.05). The results showed that the abattoir with the highest frequency of dressings per day had the higher likelihood of having contaminated chickens as compared to the abattoir with the lower frequency of dressing per day.

The number of chickens processed per day was found to be significantly associated with bacterial contamination. The odds of contamination remained the same even after controlling for training, method of washing hands, inspection of dressed chickens, and the frequency of inspections. These results are in agreement with other researchers (28, 29), who found that the cleaning time reduces as the frequency of the dressed chickens increases per day. This is because the processing plants run in more than one shift which influences the quality of the final product. The results from this study indicate that the higher the frequency of chickens dressed in a day the higher the contamination.

## Limitation and Strength of the Study

The findings of this study will help inform policy on the intervention measures used to address bacterial contamination in dressed chickens. In addition, the study findings have helped highlight the prevalence of bacterial contamination which may be useful for academic purposes, as well as in national planning.

There are some limitations in the study reported here. This study was not a quantitative study, but rather was a qualitative study that further identified and isolated both *E. coli* and *Salmonella* as these are the major indicators of micro-organism of contamination.

This study was only performed at two abattoirs because these were the only abattoirs that were receptive to this study in this area. One of the two other poultry abattoirs was being renovated and the management of the other abattoir did not permit the researchers to conduct research in their premises. This may have partly contributed to selection bias, however we believe the concept and principles applied make the findings valid.

The higher levels of bacterial contamination found after washing the carcasses points to the use of recycled water during processing and unhygienic practices indicative of poor quality control systems. This is of serious concern, as water is used to clean food surfaces, vessels, and other receptacles.

## Ethics Statement

The ethical approval to conduct this study was sought from the Excellence in Research Ethics Committee. The committee granted approval to conduct this study on bacterial contamination in abattoirs and open markets in Lusaka Province, Zambia (Ref. no. 2016-June-015). Prior to commencement of the study consent was obtained from all study participants.

## Author Contributions

PM participated in the initial conception of the study, drafted the manuscript, analysed the dataset, and carried out the statistical analysis. MM participated in the critical review of the manuscript and also helped in statistical analysis and interpretation. ARM participated in the early drafting of the manuscript and proof reading of the manuscript. JBM intellectual input for field study, manuscript development and review. JZ participated in the review of statistical analysis of results and the general review of the manuscript. All authors have read and approved the final manuscript.

### Conflict of Interest Statement

The authors declare that the research was conducted in the absence of any commercial or financial relationships that could be construed as a potential conflict of interest.
